# Downregulation of ZNF280A inhibits proliferation and tumorigenicity of colorectal cancer cells by promoting the ubiquitination and degradation of RPS14

**DOI:** 10.3389/fonc.2022.906281

**Published:** 2022-08-17

**Authors:** Binle Tian, Jingyi Zhou, Guiming Chen, Tao Jiang, Qi Li, Jian Qin

**Affiliations:** ^1^ Department of Oncology, Shanghai General Hospital, Shanghai Jiao Tong University School of Medicine, Shanghai, China; ^2^ Department of General Surgery, Shanghai General Hospital, Shanghai Jiao Tong University School of Medicine, Shanghai, China

**Keywords:** ZNF280A, RPS14, colorectal cancer, proliferation, tumorigenicity, ubiquitination

## Abstract

Colorectal cancer (CRC), one of the cancers with highest mortality, involves complicated molecular mechanisms leading to the onset of malignant phenotypes. ZNF280A, a member of the zinc-finger protein family, was shown to be a promotor of oncogenesis in CRC in this study. ZNF280A was remarkably upregulated in CRC tissues, which was meaningfully associated with tumor progression and poor prognosis in patients with CRC. Loss-of-function studies revealed that ZNF280A knockdown inhibited the development and progression of CRC as evident by the inhibition of cell proliferation, colony formation, cell apoptosis, cell cycle distribution, and cell migration *in vitro* and the repressed tumorigenesis of CRC cells *in vivo*. Next, we showed that RPS14 was the downstream target of ZNF280A and ZNF280A knockdown promoted the ubiquitination as well as degradation of RPS14 in CRC. Additionally, we demonstrated that RPS14 regulated the development of CRC *via* PI3K-Akt signaling pathway. Taken together, our findings provide a novel clear insight into ZNF280A/RPS14/PI3K-Akt axis in CRC for the first time, offering a potential target for early detection, diagnosis and treatment of CRC in future clinical applications.

## Introduction

Colorectal cancer (CRC) is the third and second most commonly diagnosed cancer in men and women, respectively (1). Up to 2021, there were 1.9 million new cases and nearly 935,000 deaths. Morbidity and mortality were significantly higher in men than in women (2). Due to the advancement of molecular biology, the researches to the mechanisms of CRC evolution became focused on the changes on gene levels in the last a few of years. Massive genetic mechanisms or protein changes involved in the development of CRC were studied and the molecular framework of CRC evolution were reported. However, the current research results are insufficient to assist effective molecular targeted therapy for CRC clinically (3). Clinical progression on CRC management can be accelerated through the identification of novel biomarkers and viable pathways for early detection and treatment of CRC.

ZNF280A is a member of the zinc-finger protein family, carrying a characteristic zinc-finger domain (4). Zinc finger proteins are the largest transcription factor family in human genome, playing a significant role in regulating and managing gene expression (5, 6). The diverse combinations and functions of zinc finger motifs make zinc finger proteins versatile in biological processes, including development, differentiation, metabolism and autophagy (6). In recent years, there have been more and more studies on the regulatory role of zinc finger proteins in human tumors (7–9). Multiple zinc finger family proteins have been found to be involved in promoting disease development in a variety of tumors (10, 11). ZNF280A has been reported to be involved in the proliferation and tumorigenicity of CRC *via* inactivating the Hippo-signaling pathway (12). However, reports on the regulatory mechanisms, biological functions and downstream targets of ZNF280A in CRC are scarce.

Ribosomal protein small subunit 14 (RPS14) is a component of the 40S ribosomal subunit and is considered to be a critical factor for ribosomal biogenesis (13). Decreased expression of RPS14 has been confirmed to be related to the 5q-syndrome in many studies (14–16). Although the role of RPS14 in hematologic malignancies has been widely reported, there are few studies investigating the association between RPS14 and solid cancers (17–19).

In this study, we demonstrated that ZNF280A was remarkably upregulated in CRC tissues, which was meaningfully associated with the tumor progression and poor prognosis in CRC patients. Loss-of-function studies revealed that ZNF280A knockdown inhibited the development and progression of CRC through the regulation of cell proliferation, colony formation, cell apoptosis, cell cycle distribution, and cell migration *in vitro* and repressed the tumorigenesis of CRC cells *in vivo*. Further researches showed that RPS14 was the downstream target of ZNF280A and knockdown of ZNF280A enhanced the ubiquitination of RPS14 in CRC. In conclusion, our study indicated that ZNF280A could be a promising novel biomarker for early detection and a potential target for blockade in CRC treatment.

## Materials and methods

### Immunohistochemistry

Human colorectal cancer and para-normal tissue chip (Cat. # HColA180Su15, Shanghai Outdo Biotech Company) was used and patients’ information was collected. For IHC staining, deparaffinized and rehydrated tissue sections were blocked and incubated with primary antibody to ZNF280A (Cat. #bs-12839R, BIOSS) and followed by incubation with a secondary antibody. DAB color was developed with diaminobenzene and hematoxylin. Slides were pictured with microscopic and viewed with ImageScope and CaseViewer. All slides were examined randomly by two independent pathologists and IHC outcomes were determined by staining percentage and intensity scores. Staining percentage scores were classified as: 1 (1–24%), 2 (25–49%), 3 (50–74%), and 4 (75%-100%). Staining intensity were scored 0 (Signalless color) to 3 (light yellow, brown and dark brown). All the antibodies used in IHC were listed in **Table S1**.

### Cell lines and cell culture

All CRC cell lines, including RKO, Caco-2, HCT116, and SW480, were obtained from Shanghai Chinese Academy of Sciences cell bank (China), and they were cultured in RPMI-1640 medium (Life Technologies, Carlsbad, CA, USA), supplemented with penicillin G (100 U/mL), streptomycin (100 mg/mL), and 10% fetal bovine serum (FBS, Life Technologies) and cultured at 37°C in a humidified atmosphere with 5% CO2.

### Real-time PCR

Total RNA from tissues or cells was extracted using TRIzol (Life Technologies), according to the manufacturer’s instructions. mRNA was polyadenylated using a poly-A polymerase-based First-Strand Synthesis kit (TaKaRa, Shanghai, China), and reverse transcription (RT) of total mRNA was performed using a PrimeScript RT Reagent kit (TaKaRa), according to the manufacturer’s protocol. cDNA was amplified and quantified on QuantStudio™ 6 Flex Real-Time PCR System (Applied Biosystems, Shanghai, China) using SYBR Green I (Applied Biosystems). The primers used in the reactions are listed in **Table S2**. Real-time PCR was performed as described previously. Glyceraldehyde-3-phosphate dehydrogenase (GAPDH) was used as the endogenous control. Relative fold expressions were calculated with the comparative threshold cycle (2^−ΔΔCt^) method according to the previous study.

### Cell transfection

For stable gene expressing, lipofectamine RNAimax (Cat. #13778-075, Thermo fisher) were used for cell RKO and HCT116 transfection with lentiviral plasmids collected. Cells were harvested after 72 h culture, and cell infection efficiency was determined using shCtrl cells as a control. The sequences used were listed in **Table S3**.

### Western blotting analysis

Cells were lysed in ice-cold radioimmunoprecipitation assay buffer (Millipore), and the protein were collected and the concentration was determined by a BCA Protein Assay Kit (HyClone-Pierce). Protein samples (20 μg per lane) were separated by 10% sodium dodecyl sulfate polyacrylamide gel electrophoresis (Invitrogen), and transferred onto polyvinylidene difluoride membranes at 4 °C. The membranes were blocked with tris-buffered saline tween-20 solution of 5% degreased milk at room temperature for 1 h and incubated with primary antibodies and GAPDH antibodies at 4 °C overnight. Then the membranes were incubated with secondary antibody HRP goat anti-rabbit IgG for 2 h at room temperature. The blots were visualized by enhanced chemiluminescence (Amersham). For Human Apoptosis Antibody Array, briefly, 20 µg total proteins were cultured with the antibody-coated array membranes and then continuing incubated with HRP linked Streptavidin conjugate. All the antibodies used in western blotting were listed in **Table S1**.

### MTT assay

The cell viability was determined by MTT assay, briefly, transfected RKO and HCT116 cells were stained with MTT reagent (5 mg/mL, GenView) and Formazan was dissolved by DMSO solution. The absorbance values at 490 nm were measured by microplate reader (Tecan) and the reference wavelength was 570 nm.

### Celigo cell counting assay

Cell proliferation rate was analyzed by Celigo cell counting assay. In brief, targeting cells were seeded at a 96-well plate with 2000 cells per well. The plate was continuously detected by Celigo (Nexcelom) for 5 days at the same time.

### Colony formation assay

For colony formation assay, cells in the logarithmic growth phase were seeded into 6-well plates in triplicate and further cultured for 8 days. Cell clones were fixed with 4% paraformaldehyde and stained with Giemsa. Then clones were photographed under a fluorescence microscope (Olympus) and colony number (clone contains >50 cells) was counted.

### Cell apoptosis and cells cycle assay

The flow cytometric methods of identifying apoptotic cells were applied using Annexin V-APC Apoptosis kit (Cat. #88–8007, eBioscience). For cells cycle assay, cells were stained with 1 mL propidium iodide (PI) staining solution (40× PI, 2 mg/mL: 100 × RNase, 10 mg/mL: 1× PBS = 25:10:1000). FACScan and FlowJo 7.6.1 (Ashland) was used for analyze. Cell apoptosis was measured and the percentage of the cells in G0-G1, S, and G2-M phase were counted and compared.

### Animal experiment

The indicated stable cell lines (1 × 10^6^) were subcutaneously injected into the right flank of 10 BALB/c (nu/nu) mice in each group. Tumor size was measured once per week and mice were sacrificed to analyze the tumor burden after 3 weeks and the tumor volume was calculated with the following formula: V =  (length × width^2^)/2. All procedures of animal experiments were performed in accordance with The Animal Care and Use Committee of Shanghai Jiao Tong University School of Medicine.

### Transwell cell invasion assay

For invasion assays, we used 8-μm filter-insert chambers (Millipore) Cells (4 × 10^4^) in 100 μl of serum-free medium were placed in the upper chamber, which had been coated with 100 μl of Matrigel (BD Biosciences), and 0.7 ml of medium containing 10% FBS was placed in the lower chamber. After incubation for 24 h, cells on the upper side of the filters were wiped off with cotton-tipped swabs, and the filters were washed with PBS. Then, the cells were fixed in 2.5% glutaraldehyde for 15 min and stained with 0.5% crystal violet for 15 min. Cells on the lower side of the filters were viewed and counted under a microscope.

### Co-immunoprecipitation

Co-immunoprecipitation from the concentrated culture medium was carried out with Dynabeads (Invitrogen) as described by the manufacturer with slight modifications. To prepare the beads for immunoprecipitation, a 40-μL bead slurry was washed twice with 200 μL PBS containing 0.005% P20 (PBSP). The antibody (Sigma) was captured on the beads by resuspending the beads in 40 μL PBSP containing 6 μL antibody and washing once in 200 μL PBSP to remove unbound antibody. Subsequently, the beads were washed twice in 200 μL PBS without P20 to wash out the P20 detergent, which may interfere with the nano-disc structure.

### Statistical analysis

All values are presented as means ± SD. Significant differences were determined using GraphPad 8.0 software (USA). Student’s t test was used to determine statistical differences between two groups. One-way ANOVA was used to determine statistical differences among multiple testing. The chi-square test was used to analyze the relationship between ZNF280A expression and clinicopathological characteristics. Survival curves were plotted using the Kaplan-Meier method and compared by log-rank test. p < 0.05 was considered significant. All the experiments were repeated three times.

## Results

### ZNF280A is highly expressed in CRC tissues

**Figure 1:**
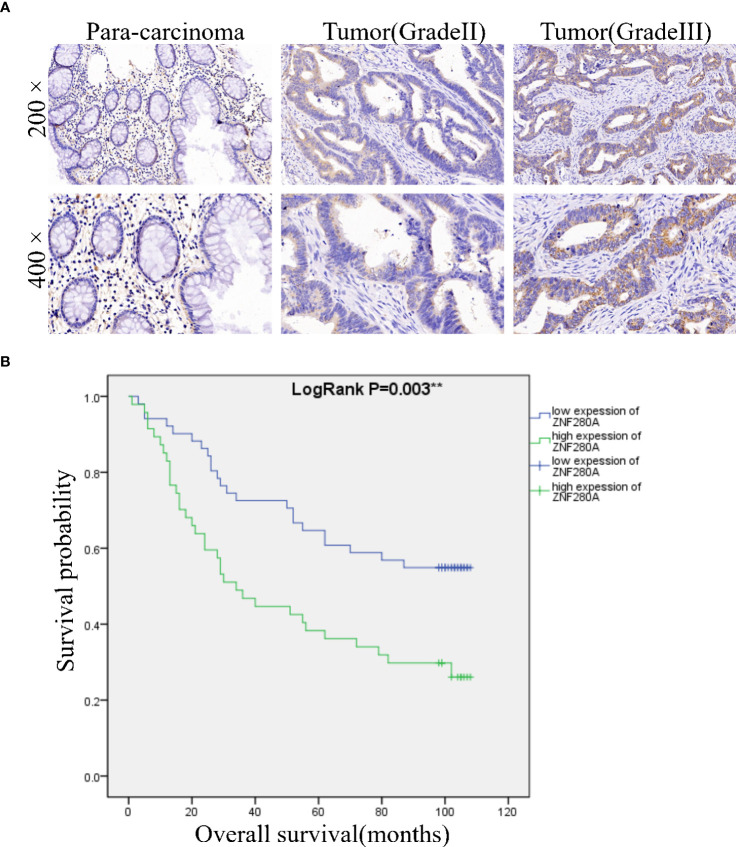
ZNF280A is highly expressed in CRC tissues. **(A)** The expression of ZNF280A in CRC tissues and para-carcinoma tissue was detected by IHC analysis. Scale bar: 50 μm. **(B)** The Kaplan–Meier survival analysis showed a significant association between ZNF280A high expression and shorter survival period of CRC patients. **P < 0.01.

Immunohistochemistry (IHC) analysis of CRC (n=98) and para-carcinoma (n=72) tissues showed the presence of ZNF280A in CRC tissues, and further manifested the higher level of ZNF280A in CRC tissues relative to that in para-carcinoma tissue (**Figure 1A**, **Table 1**). Next, we investigated the correlation between the expression of ZNF280A and clinical parameters of patients with CRC. As shown in **Table 2** and **Table S4**, high level of ZNF280A was positively correlated with clinical characteristics including pathological stage (P = 0.010) and lymphatic metastasis (P = 0.004). Moreover, Kaplan–Meier survival analysis showed the correlation between high ZNF280A expression and relatively lower survival rate (**Figure 1B**).

**Table 1:** Expression of ZNF280A in colorectal cancer tissues and para-carcinoma tissues in immunohistochemistry.

ZNF280A expression	Tumor tissue		Para-carcinoma tissue		p value
	Cases	Percentage	Cases	Percentage	
Low	51	52.0%	72	100%	0.0001***
High	47	48.0%	0	–	

**Table 2:** Correlation of ZNF280A expression with clinical pathological characteristics in patients with colorectal cancer.

Features	No. of patients	ZNF280A expression		p value
		low	high	
All patients	98	51	47	
Age (years)				0.409
≤71	47	26	21	
>71	45	21	24	
Gender				0.386
Male	53	30	23	
Female	44	21	23	
Tumor size				0.846
≤5cm	47	24	23	
>5cm	49	26	23	
Grade				0.322
II	51	29	22	
III	47	22	25	
Stage				0.010*
1	5	3	2	
2	53	34	19	
3	36	13	23	
4	3	1	2	
T Infiltrate				0.085
T1	1	0	1	
T2	5	4	1	
T3	75	42	33	
T4	13	4	9	
lymphatic metastasis (N)				0.004**
N0	58	37	21	
N1	27	10	17	
N2	11	3	8	
Lymph node positive				0.009**
≤0	48	30	18	
>0	38	13	25	

### ZNF280A knockdown inhibits CRC progression *in vitro*

**Figure 2:**
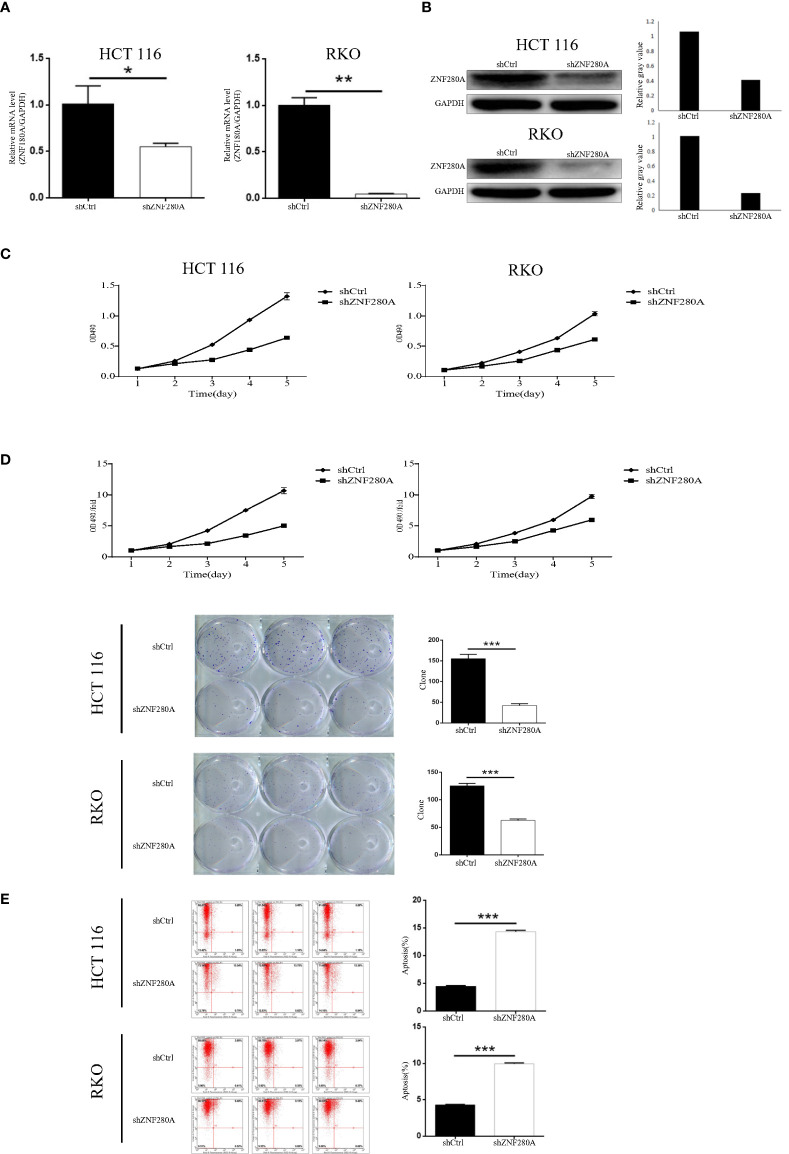
ZNF280A knockdown inhibits CRC progression *in vitro.* **(A, B)** Real-time PCR **(A)** and western blotting **(B)** analyses of ZNF280A expression in the indicated CRC cells. GAPDH was used as endogenous control in western blot. **(C)** MTT assay revealed that knockdown of ZNF280A suppressed the proliferation of HCT-116 and RKO cells. **(D)** Colony formation assay showed that knockdown of ZNF280A inhibited the colony-forming ability of HCT-116 and RKO cells. **(E)** Apoptosis of HCT-116 and RKO cells with or without ZNF280A knockdown were determined by flow cytometry. Data were shown as mean ± SD. *P < 0.05, **P < 0.01, ***P < 0.001.

We first engineered HCT116 and RKO cells that stably silenced ZNF280A by endogenously knocking down ZNF280A *via* lentivirus infection, and subsequently evaluated the efficiency of transfection by fluorescence imaging (**Figure S1**), and assessed the knockdown efficiency by qPCR and western blotting (**Figures 2A, B**). MTT (3- (4,5-dimethylthiazol-2-yl)-2,5-diphenyltetrazolium bromide) assay was first carried out to investigate the regulatory role of ZNF280A on HCT116 and RKO cells. The result showed that knockdown of ZNF280A markedly decreased the proliferation of CRC cells (**Figure 2C**). Colony formation assays indicated that knockdown of ZNF280A repressed the colony-generating capability of both HCT116 and RKO cells (**Figure 2D**). The results of flow cytometric analysis showed the enhanced cell apoptosis of shZNF280A cells (**Figure 2E**). Collectively, the results demonstrated that knock down of ZNF280A inhibited the capability of tumorigenesis of CRC cells *in vitro*, supporting the findings from immunohistochemical analysis of CRC tissues.

### ZNF280A knockdown inhibits the tumorigenesis of CRC *in vivo*

**Figure 3:**
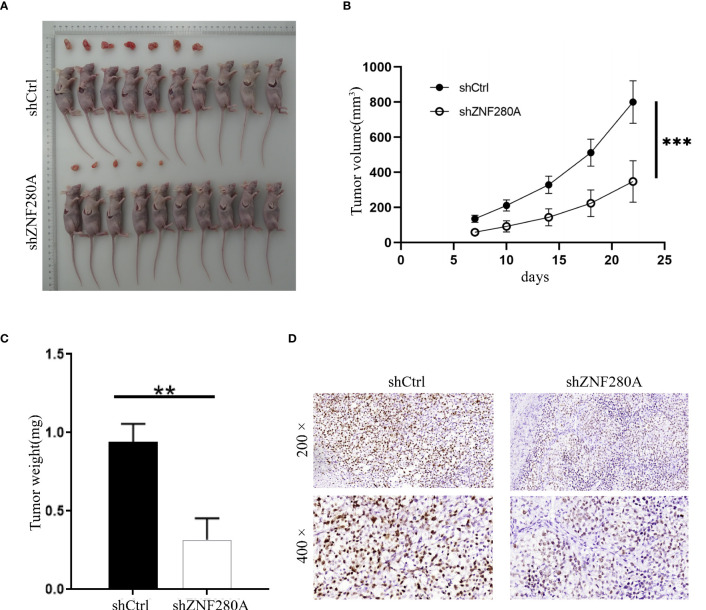
ZNF280A knockdown inhibits the tumor tumorigenesis of CRC in vivo. **(A)** Photos of tumors removed from the BALB/c mice at day 35 after the injection of RKO cells with or without ZNF280A knockdown. **(B)** Tumor volumes of mice in shZNF280A and shCtrl groups were measured. **(C)** The excised tumors from the mice were weighed. **(D)** The expression levels of Ki67 in the sections sliced from the excised tumors were detected by IHC staining. Scale bar: 50 μm. Data were shown as mean 1 ± 2 SD. **P < 0.01, ***P < 0.001.

The effects of ZNF280A on the tumorigenesis of CRC were further investigated *in vivo*. Mice were randomly divided into two groups (n = 10/group), and 1 × 10^6^ RKO cells with or without ZNF280A knockdown were inoculated subcutaneously into the mice in each group, respectively. The results showed (**Figure 3A–C**) that the volumes and weights of tumor in the mice injected with shZNF280A RKO cells were remarkably decreased as compared to those of the shCtrl group. Consistently, ZNF280A knockdown decreased the numbers of Ki67-positive staining cells, reflecting hypo-proliferative activity of these cells (**Figure 3D**). Thus, these results demonstrated that shZNF280A significantly suppressed the tumorigenicity of CRC *in vivo*.

### RPS14 is the downstream target of ZNF280A in the regulation of CRC

**Figure 4:**
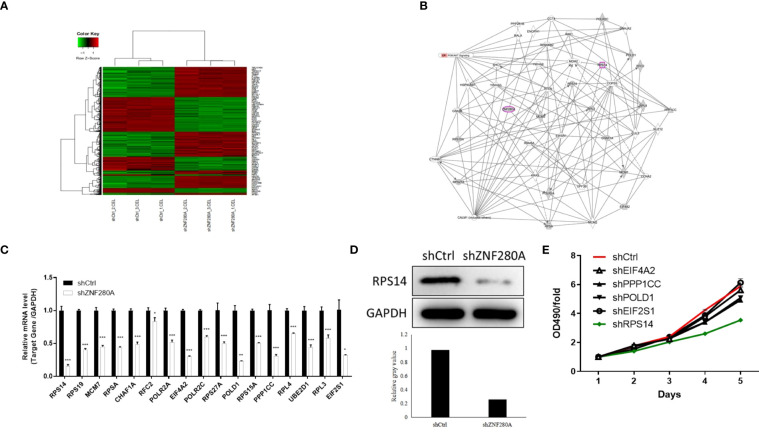
RPS14 is the downstream target of ZNF280A in the regulation of CRC. **(A)** A PrimeView Human Gene Expression Array was performed to identify the DEGs between shZNF280A and shCtrl groups of RKO cells. **(B)** A ZNF280A associated interaction network constructed by IPA analysis revealed the potential linkage between ZNF280A and RPS14. **(C–E)** Real-time PCR **(C)**, western blotting **(D)** and MTT **(E)** analyses were used to detect the expression of several selected DEGs in RKO cells with or without ZNF280A. *P < 0.05, **P < 0.01, ***P < 0.001.

To explore the downstream target of ZNF280A in the regulation of CRC, microarray analysis of RKO cells with or without ZNF280A identified 7099 differentially expressed genes (DEGs), including 4029 upregulated ones and 3070 downregulated ones (**Figures 4A**, **S2A**). The quality of all the microarray data was assessed in several ways (**Figures S2B–E**). Hence, the enrichment of all DEGs in canonical signaling pathways and disease/function was interpreted by ingenuity pathway analysis (IPA), showing cell cycle control of chromosomal replication as one of the most enriched pathways and cancer as one of the most enriched diseases (**Figures S2F, G**). Furthermore, based on all above results derived from a combination of the molecular interaction networks constructed central to ZNF280A, some DEGs were identified to be promising downstream events of ZNF280A (**Figure 4B**). Subsequently, a variety of top-ranked DEGs was subject to qPCR, western blotting and MTT assay for the assessment of differential expression regulated by ZNF280A (**Figures 4C–E**). Collectively, the results revealed that RPS14 could be the most promising downstream target of ZNF280A.

### ZNF280A knockdown alleviates the growth of CRC induced by RPS14 overexpression

**Figure 5:**
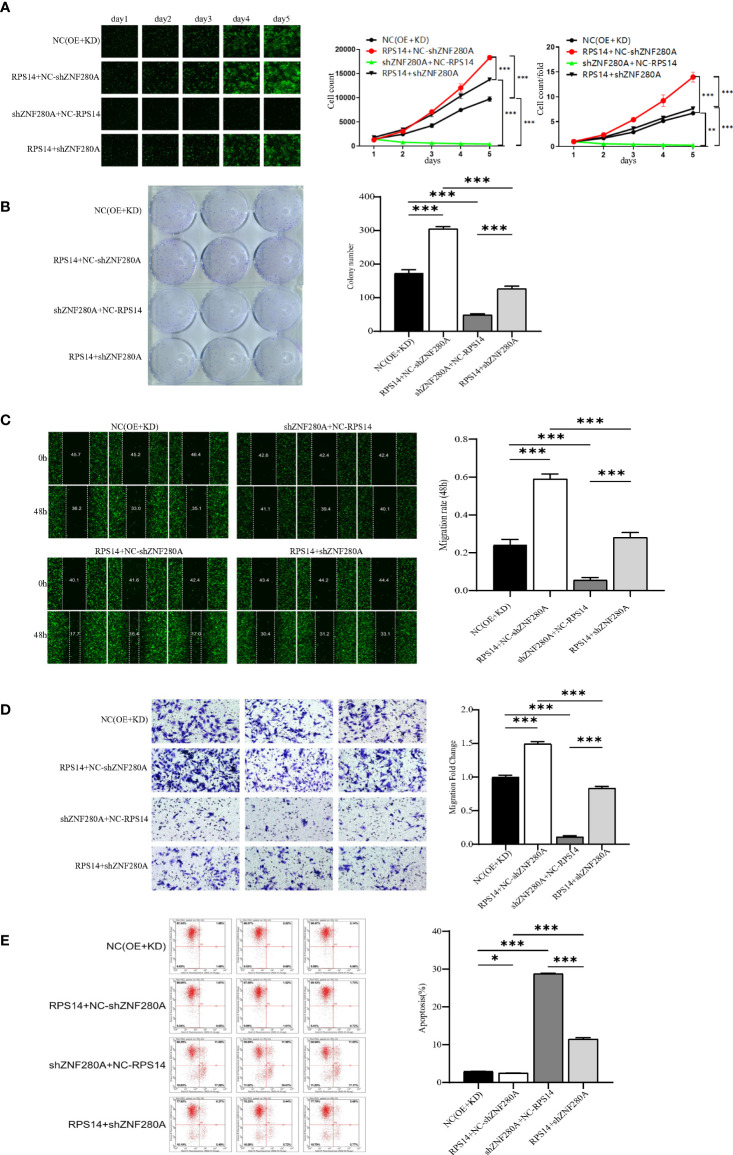
ZNF280A knockdown alleviates RPS14 overexpression induced promotion of CRC **(A–E)** RKO cells transfected by NC (OE + KD), RPS14 overexpression lentivirus, ZNF280A knockdown lentivirus, and simultaneous RPS14 overexpression and ZNF280A knockdown lentivirus were subject to the detection of cell proliferation by Celigo cell counting assay **(A)**, colony formation **(B)**, cell migration by wound-healing assay **(C)** and cell migration by Transwell assay **(D)**, and cell apoptosis by flow cytometry **(E)**. The representative images were selected from at least three independent experiments. Data were shown as mean ± SD. *P < 0.05, **P < 0.01, ***P < 0.001.

In order to further clarify the effects of ZNF280A/RPS14 axis in the regulation of CRC, we analyzed the expression of RPS14 in CRC cells by qPCR (**Figure S3A**), and constructed RPS14-overexpressed cells, shZNF280A cells, as well as cells with simultaneous RPS14 overexpression and ZNF280A knockdown (**Figure S3B** for transfection efficiency, **Figures S3C, D** for expression detection) *via* lentiviral infection. As results (**Figure 5**), RPS14 overexpression promoted the development of CRC through a combination of effects which includes promoting cell proliferation (**Figure 5A**), enhancing colony formation (**Figure 4B**), inhibiting cell migration (**Figures 5C, D**), and suppressing cell apoptosis (**Figure 5E**). On the other hand, the overall inhibitory effects of cell growth (**Figure 5A**), colony formation (**Figure 5B**), suppression of cell migration (**Figure 5C, D**), and increase of cell apoptosis (**Figure 5E**) warranted that the effects of ZNF280A knockdown on CRC development could be attenuated or reversed by the overexpression of RPS14, highlighting the role of ZNF280A/RPS14 axis in CRC.

### RPS14 regulates the development of CRC *via* PI3K-Akt signaling pathway

**Figure 6:**
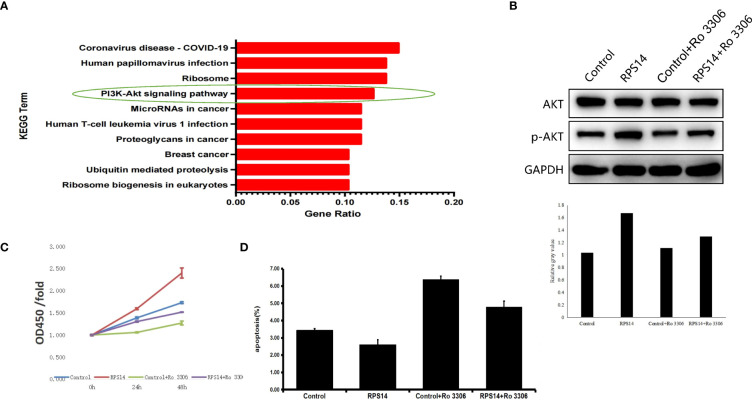
RPS14 plays a role in CRC via PI3K-Akt signaling pathway. **(A)** Gene-set enrichment analysis of RPS14 by KEGG was performed. **(B)** Western blotting was used to assess the phosphorylation level of Akt in cells treated by selective CDK1 inhibitor (Ro-3306) with or without RPS14 overexpression. **(C, D)** CCK-8 (C) and flow cytometry **(D)** were used to detect the effects of Ro-3306 on cell growth and apoptosis with or without RPS14 overexpression. Data were shown as mean ± SD.

To explore the underlying signaling pathway mediating the effects of RPS14 on development of CRC, gene-set enrichment analysis of RPS14 by Kyoto Encyclopedia of Genes and Genomes (KEGG) showed PI3K-Akt signaling pathway as one of the most enriched pathways (**Figure 6A**). Furthermore, Western blotting was used to assess the phosphorylation level of Akt in cells treated by selective CDK1 inhibitor (Ro-3306) with or without RPS14 overexpression (**Figure 6B**). Cell counting kit-8 (CCK-8) and flow cytometry were employed to show the effects of Ro-3306 on cell growth and apoptosis with or without RPS14 overexpression (**Figures 6C, D**). These results demonstrated that the influence of RPS14 overexpression on the phosphorylation level of Akt and development in CRC cells could be alleviated by CDK1 inhibitors, revealing that RPS14 exerted oncogenic function *via* PI3K-Akt signaling pathway.

### ZNF280A knockdown promotes the ubiquitination and degradation of RPS14

**Figure 7:**
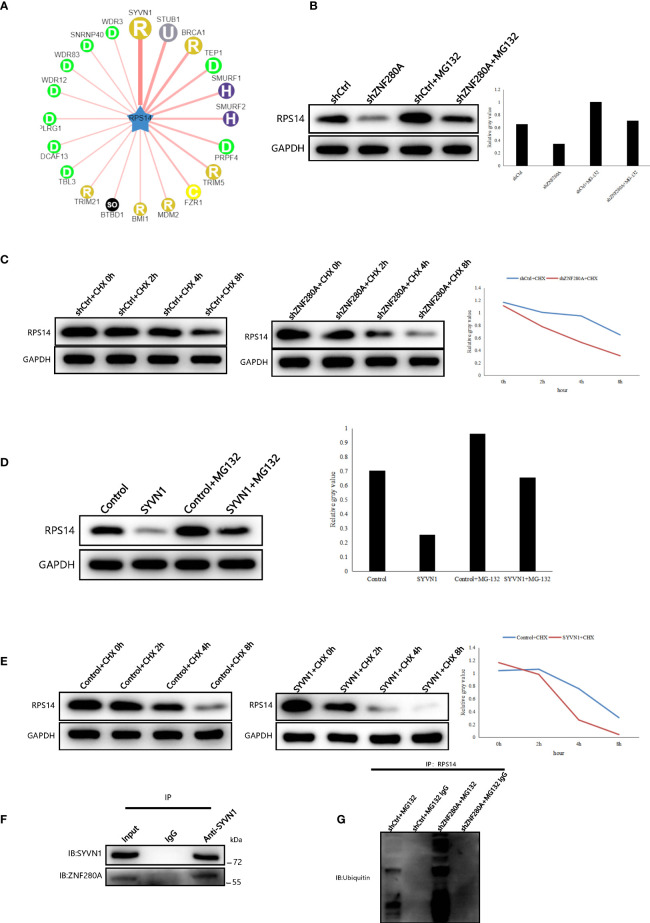
ZNF280A knockdown promotes the ubiquitination and degradation of RPS14 **(A)** Network view of predicted E3 ubiquitin ligases in UbiBrowser web services. In network view, the central node is the queried E3 substrate, and the surrounding nodes are the predicted ubiquitin ligases. The width of the edge reflects the confidence of the interaction. **(B–E)** Western blotting of RPS14 expression in the indicated cells. **(F, G)** Co-immunoprecipitation was applied to detect the interaction of SYVN1 and ZNF280A **(F)**, as well as the regulation of ZNF280A on ubiquitination of RPS14 **(G)**.

To identify potential molecular mechanism influencing the regulation of ZNF280A on RPS14 in CRC cells, we queried the E3 ubiquitin ligases of RPS14 in the web tool of UbiBrowser (20). In total, 6 predicted E3 ligases with middle-confidence interactions and 36 predicted E3 ligases with low-confidence interactions were determined (**Table S5**). Some of the predicted E3 ligases were displayed (**Figure 7A**). Moreover, subcellular localization analysis indicated that there was a great possibility of interaction between ZNF280A and SYVN1 (**Figure S4**). Subsequently, we found that the expression of RPS14 in RKO cells could be inhibited by ZNF280A knockdown and this inhibition could be reversed by proteasome inhibitor MG132 (**Figure 7B**). In addition, the degradation of RPS14 was dramatically accelerated by ZNF280A knockdown (**Figure 7C**). Interestingly, the E3 ubiquitin ligase SYVN1 exhibited a similar function with ZNF280A knockdown (**Figures 7D, E**). The endogenous interaction of SYVN1 and ZNF280A was further demonstrated by the co-immunoprecipitation assay, which may be an explanation of the ZNF280A-induced regulation of RPS14 stability (**Figure 7F**). Finally, we confirmed that ZNF280A knockdown promoted the ubiquitination of RPS14 (**Figure 7G**). Therefore, the regulatory mechanism of ZNF280A on RPS14 may be related to ubiquitin-proteasome system.

## Discussion

CRC is a globally prevalent and highly aggressive malignancy. Despite the tremendous efforts in the treatment of CRC, clinical outcomes remain bleak. A mass of molecules and relevant signaling pathways in CRC cells are of great significance to the malignant phenotypes of CRC (21). Hence, a better understanding of the complex signaling pathways in CRC is urgently required to elucidate the underlying mechanism of CRC progression and pharmacotherapy. The high expression of ZNF280A in CRC was recently reported to be associated with proliferation and tumorigenesis of cancer cells *via* inactivating the Hippo-signaling pathway (12).

In the current study, we first demonstrated the expression pattern of ZNF280A in CRC tissues, and we observed that ZNF280A was upregulated in CRC tissues compared to adjacent tissues, which played a crucial role in CRC tumorigenesis. Then, we found that ZNF280A knockdown repressed proliferation and enhanced apoptosis *in vitro*, and it inhibited tumorigenesis *in vivo*. Furthermore, we detected the gene expression profile of CRC cells with or without ZNF280A by bioinformatics analysis and suspected that RPS14 may be a downstream event of ZNF280A in CRC development. Subsequent results showed that ZNF280A knockdown alleviated the promotion of CRC induced by RPS14 overexpression, indicating RPS14 as the downstream event of ZNF280A in CRC development. In order to explore for the possible regulatory mechanism of ZNF280A on RPS14 in CRC cells, we applied the web tool of UbiBrowser to forecasted the E3 ubiquitin ligases of RPS14. We deduced that SYVN1 may be the most possible E3 ubiquitin ligases of RPS14 based on the results of UbiBrowser and subcellular localization analysis. Hence, we verified the regulatory mechanism of ZNF280A on ubiquitination and degradation of RPS14. In addition, our results also proved that RPS14 functioned *via* PI3K-Akt signaling pathway in CRC. Therefore, our findings showed that RPS14 was a downstream molecule of ZNF280A in the regulation of CRC development, and revealed how ZNF280A regulated the expression of RPS14.

As the largest transcription factor family, Zinc finger proteins play crucial roles in the regulation of gene expression (5, 6). However, our understanding about the biological role of ZNF280A in cancers is far from enough. The correlation between ZNF280A and hematologic malignancies has been previously reported (22–24). Their studies showed that the function of ZNF280A was correlated with deletions of chromosome 22q11. However, the functional mechanism and clinical significance of ZNF280A were not explored in depth. Recently, Wang et al. reported that ZNF280A was involved in the proliferation and tumorigenicity of CRC *via* inactivating the Hippo-signaling pathway, and Liu et al. found the promotion effect of ZNF280A in the development of lung adenocarcinoma by regulating the expression of EIF3C (4, 12). Our findings revealed a novel mechanism that ZNF280A promoted the tumorigenesis of CRC by attenuating the ubiquitination and degradation of RPS14.

RPS14 is considered to be associated with the 5q-syndrome and some kinds of hematologic malignancies (14, 17). In solid tumors, some bioinformatics analyses found the association between RPS14 and triple-negative breast cancer, as well as bladder cancer (25, 26). The oncogenic role of RPS14 was further determined regarding the proliferation and metastasis of estrogen receptor-positive breast cancer cells (27). Additionally, RPS14 was identified as one of the reference genes for qRT-PCR in lung squamous-cell carcinoma by RNA-Seq (28). No other reports about the function of RPS14 in solid tumor can be found so far. In this study, we explored the upstream molecule of RPS and elucidated the regulatory mechanism of ZNF280A on RPS14 in CRC. Besides, our findings proved that RPS14 functioned *via* PI3K-Akt signaling pathway in CRC.

In conclusion, we showed the expression of ZNF280A in CRC was high and explored its regulatory mechanism. We also demonstrated the regulation of ZNF280A on ubiquitination and degradation of RPS14 and the downstream signaling of RPS14 *via* PI3K-Akt signaling pathway. Accordingly, our findings provide a novel clear insight about ZNF280A/RPS14/PI3K-Akt axis in CRC for the first time, offering a potential target for early detection, diagnosis and treatment of CRC in future clinical applications.

## Data availability statement

The datasets presented in this study can be found in online repositories. The names of the repository/repositories and accession number(s) can be found below: https://github.com/tianbinle/Data.

## Ethics statement

The studies involving human participants were reviewed and approved by Medical Experiment Ethics Committee at Shanghai General Hospital affiliated to Shanghai Jiao Tong University in accordance with the Declaration of Helsinki. The patients/participants provided their written informed consent to participate in this study. The animal study was reviewed and approved by Committees of Shanghai Jiao Tong University, Shanghai, China. Written informed consent was obtained from the individual (s) for the publication of any potentially identifiable images or data included in this article.

## Author contributions

JQ and QL conceived the project and designed the studies. BT and JZ performed the experiments. GC and TJ analyzed the data. BT wrote the paper. JQ and QL revised the manuscript. All authors read and approved the final paper. All authors contributed to the article and approved the submitted version.

## Funding

This work was supported by the National Natural Science Foundation of China (No. 81370561; NO.82073214), Outstanding disciplines leaders of Shanghai Municipal Commission of Health and Family Planning (NO. 2018BR39) and Bethune Charitable Foundation.

## Conflict of interest

The authors declare that the research was conducted in the absence of any commercial or financial relationships that could be construed as a potential conflict of interest.

## Publisher’s note

All claims expressed in this article are solely those of the authors and do not necessarily represent those of their affiliated organizations, or those of the publisher, the editors and the reviewers. Any product that may be evaluated in this article, or claim that may be made by its manufacturer, is not guaranteed or endorsed by the publisher.

## Supplementary material

Supplementary Figure 1 The transfection efficiencies of shZNF280A and shCtrl in HCT116 and RKO cells were evaluated through observing the fluorescence of GFP on lentivirus vector.

Supplementary Figure 2
**(A)** The volcano plot of gene expression profiling in RKO cells with or without ZNF280A knockdown. Green dots represented the downregulated DEGs, red dots represented the upregulated DEGs. **(B–E)** The quality of all the microarray data was assessed in several ways. **(F, G)** Results showed cell cycle control of chromosomal replication as one of the most enriched pathways and cancer as one of the most enriched diseases.

Supplementary Figure 3
**(A)** The expression of RPS14 in CRC cells was analyzed by qPCR. **(B)** The transfection efficiencies in RKO cells were evaluated through observing the fluorescence of GFP on lentivirus vector. **(C, D)** The expression of ZNF280A and RPS14 of the indicated cells were e analyzed by qPCR and WB. *P < 0.05, **P < 0.01, ***P < 0.001.

Supplementary Figure 4 Subcellular localization analysis indicated that there was a great possibility of interaction between ZNF280A and SYVN1.
