# Supplementary material for: Downregulation of ZNF280A inhibits proliferation and tumorigenicity of colorectal cancer cells by promoting the ubiquitination and degradation of RPS14
Source: Front Oncol. 2022 Aug 17;12:906281. doi: 10.3389/fonc.2022.906281 (PMC9428494; doi:10.3389/fonc.2022.906281)
Supplement: Supplementary file 1 [file Table_1.docx]

Table S1 Antibodies used in western blotting and IHC

| Primary antibodies | kDa | Dilution in WB | | Source species | | | Company | | Catalog No. | |  |
| --- | --- | --- | --- | --- | --- | --- | --- | --- | --- | --- | --- |
| GAPDH | 36 | 1:30000 | | Mouse | | | Proteintech | | 60004-1-lg | |  |
| ZNF280A | 61 | 1:1000 | | Mouse | | | abcam | | ab169117 | |  |
| RPS14 | 16 | 1:500 | | Rabbit | | | abcam | | ab246916 | |  |
| ZNF280A | 50 | 1:1000 | | Rabbit | | | Absin | | abs140559 | |  |
| AKT | 60 | 1:3000 | | Rabbit | | | CST | | 4691S | |  |
| p-AKT | 56 | 1:1000 | | Rabbit | | | Abcam | | ab38449 | |  |
| SYVN1 | 68 | 1:2000 | | Rabbit | | | Proteintech | | 13473-1-AP | |  |
| Ubiquitin | 28 | 1:1000 | | Mouse | | | Santa Cruz | | sc-47721 | |  |
| Primary antibodies | kDa | | Dilution in IHC | | Source species | | | Company | | Catalog No. |  |
| ZNF280A | 61 | | 1:200 | | Rabbit | | | abcam | | ab169117 |  |
| Ki67 | 55 | | 1:100 | | Rabbit | | | abcam | | ab16667 |  |
| Secondary antibody | Dilution | | | | | Company | Catalog No. | | | | |
| HRP Goat Anti-Rabbit IgG (WB) | 1:3000 | | | | | Beyotime | A0208 | | | | |
| HRP Goat Anti-Mouse IgG (WB) | 1:3000 | | | | | Beyotime | A0216 | | | | |
| HRP Goat Anti-Rabbit IgG (IHC) | 1:400 | | | | | abcam | ab97080 | | | | |
