# Supplementary material for: Downregulation of ZNF280A inhibits proliferation and tumorigenicity of colorectal cancer cells by promoting the ubiquitination and degradation of RPS14
Source: Front Oncol. 2022 Aug 17;12:906281. doi: 10.3389/fonc.2022.906281 (PMC9428494; doi:10.3389/fonc.2022.906281)
Supplement: Supplementary file 2 [file Table_2.docx]

Table S2 Primers used in qPCR

| Gene | Forward primer sequence (5’-3’) | Reverse primer sequence (5’-3’) | bp |
| --- | --- | --- | --- |
| GAPDH | TGACTTCAACAGCGACACCCA | CACCCTGTTGCTGTAGCCAAA | 121 |
| ZNF280A | GATCTGATCTATGTTGGGGTGGA | CGTGAGCAGGATATTGACGGA | 166 |
| RPS14 | GAAAGGGGAAGGAAAAGAAGG | TGCAAAGATATGGCAGACACC | 95 |
| RPS19 | CGAATGGGTGGATACCGTCA | TCACCAGCTCGCGTGTAGAA | 84 |
| MCM7 | ATCACGGTGCTGGTAGAAGG | CGCAGGATTGGCAAGAAA | 89 |
| RPSA | GCTGAGAAGGCAGTGACCAA | TGCTGAATAGGCACAGAGGG | 131 |
| CHAF1A | CAGTGATGTCGTCATCGTGG | TGAGTGCCGTCTTCTTATTCC | 140 |
| RFC2 | GCTTCGCCCTTGCTTGTAA | CTTCTAGGCCGTCATCAGTGTA | 162 |
| POLR2A | AGCTGTATGCGGAATGGAAG | CCTCATCTGAGATGCGTTTGA | 105 |
| EIF4A2 | TTTCTGCCACAATGCCAACT | CCTTCAAGGGTCAATTCTTCCT | 100 |
| POLR2C | CTGGGCGAGATGCCGTAC | AACCTCAGCGATGAAGACC | 135 |
| RPS27A | TTGAGACTTCGTGGTGGTGC | TTGCCATAAACACCCCAGCA | 190 |
| POLD1 | TGTTACACCACGCTCCTTCG | AGGTCTTCACAAACTCGTCCC | 100 |
| RPS15A | CGCGCCGCCACAATG | CACAGTGAGAAACCGGACGA | 132 |
| PPP1CC | AGGCGATGGCGGATTTAG | CATTCTTACCAGGCTTGGAC | 87 |
| RPL4 | CCGTTGGCATCGTAGAGTGA | AGACATGACCAGTGCTGGTAGG | 91 |
| UBE2D1 | ATAGCGCATATCAAGGTGGA | GTGAAAGCAATCTTTGGTGG | 85 |
| RPL3 | AGAGGCTTGAGCAGCAGGTA | ACGACTGGTGACCCCTTTGT | 107 |
| EIF2S1 | ACCTGGATATGGTGCCTATGAT | GAATTTTGACAGCCTGTGGG | 143 |
|  |  |  |  |
