# Supplementary material for: Downregulation of ZNF280A inhibits proliferation and tumorigenicity of colorectal cancer cells by promoting the ubiquitination and degradation of RPS14
Source: Front Oncol. 2022 Aug 17;12:906281. doi: 10.3389/fonc.2022.906281 (PMC9428494; doi:10.3389/fonc.2022.906281)
Supplement: Supplementary file 3 [file Table_3.docx]

Table S3 The target sequences and shRNA sequences

| Gene | No. | Target sequence (5'-3') | shRNA sequences (5'-3') |
| --- | --- | --- | --- |
| ZNF280A | Pbr10330-a | CTGTCACTATGAAGTCTTCAT | ccggCTGTCACTATGAAGTCTTCATctcgagATGAAGACTTCATAGTGACAGtttttg |
| ZNF280A | Pbr103300-b | CTGTCACTATGAAGTCTTCAT | aattcaaaaaCTGTCACTATGAAGTCTTCATctcgagATGAAGACTTCATAGTGACAG |
