# Supplementary material for: Downregulation of ZNF280A inhibits proliferation and tumorigenicity of colorectal cancer cells by promoting the ubiquitination and degradation of RPS14
Source: Front Oncol. 2022 Aug 17;12:906281. doi: 10.3389/fonc.2022.906281 (PMC9428494; doi:10.3389/fonc.2022.906281)
Supplement: Supplementary file 4 [file Table_4.docx]

Table S4. Relationship between ZNF280A expression and tumor characteristics in patients with colorectal cancer analyzed by Spearman rank correlation analysis.

|  |  | ZNF280A |
| --- | --- | --- |
| Stage | Pearson correlation | 0.259 |
|  | Significance (two tailed) | 0.010* |
|  | N | 97 |
| Lymph node positive | Pearson correlation | 0.281 |
|  | Significance (two tailed) | 0.009** |
|  | N | 86 |
| [lymphatic](D:/360%E5%AE%89%E5%85%A8%E6%B5%8F%E8%A7%88%E5%99%A8%E4%B8%8B%E8%BD%BD/Dict/8.4.0.0/resultui/html/index.html#/javascript:;) [metastasis](D:/360%E5%AE%89%E5%85%A8%E6%B5%8F%E8%A7%88%E5%99%A8%E4%B8%8B%E8%BD%BD/Dict/8.4.0.0/resultui/html/index.html#/javascript:;)（N） | Pearson correlation | 0.295 |
|  | Significance (two tailed) | 0.004** |
|  | N | 96 |
