# Supplementary material for: Downregulation of ZNF280A inhibits proliferation and tumorigenicity of colorectal cancer cells by promoting the ubiquitination and degradation of RPS14
Source: Front Oncol. 2022 Aug 17;12:906281. doi: 10.3389/fonc.2022.906281 (PMC9428494; doi:10.3389/fonc.2022.906281)
Supplement: Supplementary file 5 [file Table_5.docx]

Table S5. The ligases were predicted by using UbiBrowser.

| E3 | E3GENE | SUB | SUBGENE | HOMO | PFAM | GO | NET | MOTIF | SCORE |
| --- | --- | --- | --- | --- | --- | --- | --- | --- | --- |
| Q86TM6 | SYVN1 | P62263 | RPS14 | 1 | 1 | 1.25 | 1.44 | 6.61 | 0.746 |
| Q9UNE7 | STUB1 | P62263 | RPS14 | 1 | 1 | 3.77 | 2.39 | 1 | 0.699 |
| P38398 | BRCA1 | P62263 | RPS14 | 1 | 1 | 3.77 | 2.2 | 1 | 0.691 |
| Q99973 | TEP1 | P62263 | RPS14 | 1 | 1 | 3.77 | 1.44 | 1 | 0.676 |
| Q9HCE7 | SMURF1 | P62263 | RPS14 | 1 | 1 | 2.93 | 1.69 | 1.06 | 0.673 |
| Q9HAU4 | SMURF2 | P62263 | RPS14 | 1 | 1 | 2.93 | 2.2 | 1 | 0.667 |
| O43172 | PRPF4 | P62263 | RPS14 | 1 | 1 | 3.77 | 1.29 | 1 | 0.665 |
| Q9C035 | TRIM5 | P62263 | RPS14 | 1 | 1 | 3.77 | 1.29 | 1 | 0.665 |
| Q9UM11 | FZR1 | P62263 | RPS14 | 1 | 1 | 1.51 | 1.44 | 2.12 | 0.66 |
| Q00987 | MDM2 | P62263 | RPS14 | 1 | 1 | 2.33 | 1.69 | 1 | 0.645 |
| Q9H0C5 | BTBD1 | P62263 | RPS14 | 1 | 1 | 3.77 | 1 | 1 | 0.64 |
| Q8TEQ6 | GEMIN5 | P62263 | RPS14 | 1 | 1 | 3.77 | 1 | 1 | 0.64 |
| Q96DI7 | SNRNP40 | P62263 | RPS14 | 1 | 1 | 3.77 | 1 | 1 | 0.64 |
| Q9GZL7 | WDR12 | P62263 | RPS14 | 1 | 1 | 3.77 | 1 | 1 | 0.64 |
| Q9NV06 | DCAF13 | P62263 | RPS14 | 1 | 1 | 3.77 | 1 | 1 | 0.64 |
| Q9BRX9 | WDR83 | P62263 | RPS14 | 1 | 1 | 3.77 | 1 | 1 | 0.64 |
| O43290 | SART1 | P62263 | RPS14 | 1 | 1 | 3.77 | 1 | 1 | 0.64 |
| O43660 | PLRG1 | P62263 | RPS14 | 1 | 1 | 3.77 | 1 | 1 | 0.64 |
| O95376 | ARIH2 | P62263 | RPS14 | 1 | 1 | 1 | 1.77 | 2.12 | 0.64 |
| Q9UNX4 | WDR3 | P62263 | RPS14 | 1 | 1 | 3.77 | 1 | 1 | 0.64 |
| P19474 | TRIM21 | P62263 | RPS14 | 1 | 1 | 3.77 | 1 | 1 | 0.64 |
| Q9UMS4 | PRPF19 | P62263 | RPS14 | 1 | 1 | 3.77 | 1 | 1 | 0.64 |
| P35226 | BMI1 | P62263 | RPS14 | 1 | 1 | 2.93 | 1.29 | 1 | 0.64 |
| Q12788 | TBL3 | P62263 | RPS14 | 1 | 1 | 3.77 | 1 | 1 | 0.64 |
| Q13064 | MKRN3 | P62263 | RPS14 | 1 | 1 | 3.77 | 1 | 1 | 0.64 |
| Q86YJ5 | 9-Mar | P62263 | RPS14 | 1 | 1 | 1.25 | 1 | 2.8 | 0.633 |
| Q96PU5 | NEDD4L | P62263 | RPS14 | 1 | 1 | 1.25 | 1 | 2.8 | 0.633 |
| Q9NW38 | FANCL | P62263 | RPS14 | 1 | 1 | 1.25 | 1.29 | 2.12 | 0.63 |
| Q13309 | SKP2 | P62263 | RPS14 | 1 | 1 | 1.24 | 1.29 | 2.12 | 0.63 |
| O95630 | STAMBP | P62263 | RPS14 | 1 | 1 | 2.33 | 1.68 | 1 | 0.618 |
| Q9ULJ6 | ZMIZ1 | P62263 | RPS14 | 1 | 1 | 2.93 | 1 | 1 | 0.615 |
| Q92831 | KAT2B | P62263 | RPS14 | 1 | 1 | 1.51 | 2.39 | 1 | 0.609 |
| Q15542 | TAF5 | P62263 | RPS14 | 1 | 1 | 1.51 | 2.39 | 1 | 0.609 |
| Q96FA3 | PELI1 | P62263 | RPS14 | 1 | 1 | 1 | 1.29 | 2.12 | 0.608 |
| P22681 | CBL | P62263 | RPS14 | 1 | 1 | 1.78 | 1.44 | 1.06 | 0.607 |
| O95071 | UBR5 | P62263 | RPS14 | 1 | 1 | 1.51 | 2.3 | 1 | 0.605 |
| Q99728 | BARD1 | P62263 | RPS14 | 1 | 1 | 1.51 | 1.77 | 1 | 0.605 |
| Q96D21 | RASD2 | P62263 | RPS14 | 1 | 1 | 1.51 | 2.3 | 1 | 0.605 |
| P51948 | MNAT1 | P62263 | RPS14 | 1 | 1 | 1.51 | 1.77 | 1 | 0.605 |
| Q12834 | CDC20 | P62263 | RPS14 | 1 | 1 | 1.51 | 1.77 | 1 | 0.605 |
| Q15386 | UBE3C | P62263 | RPS14 | 1 | 1 | 1.25 | 1 | 2.12 | 0.604 |
| Q9NWF9 | RNF216 | P62263 | RPS14 | 1 | 1 | 1.25 | 1 | 2.12 | 0.604 |
