# Supplementary figures and images for: Downregulation of ZNF280A inhibits proliferation and tumorigenicity of colorectal cancer cells by promoting the ubiquitination and degradation of RPS14
Source: Front Oncol. 2022 Aug 17;12:906281. doi: 10.3389/fonc.2022.906281 (PMC9428494; doi:10.3389/fonc.2022.906281)

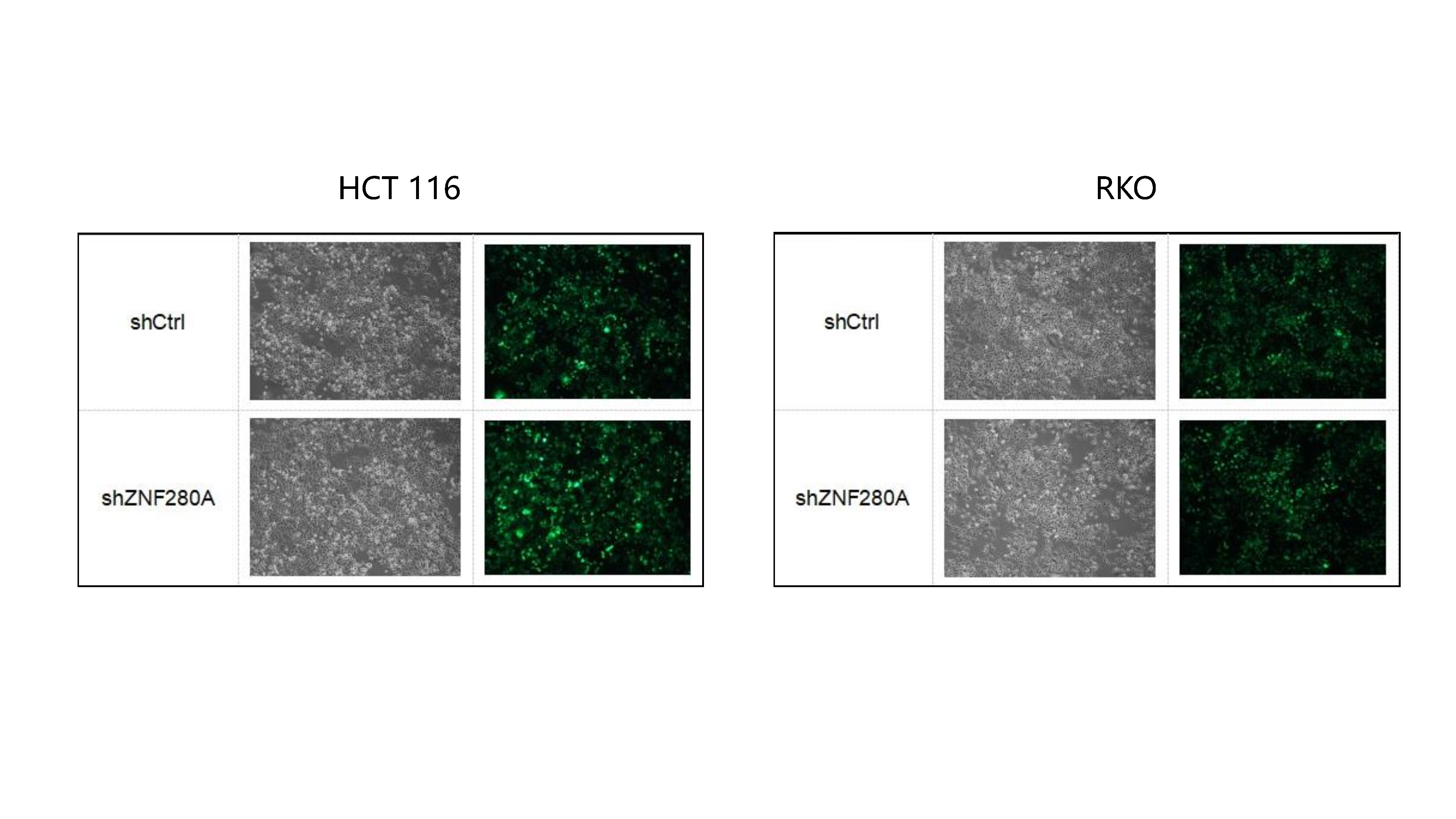

Supplement: Supplementary Figure 1 — The transfection efficiencies of shZNF280A and shCtrl in HCT116 and RKO cells were evaluated through observing the fluorescence of GFP on lentivirus vector. [file Image_1.png]

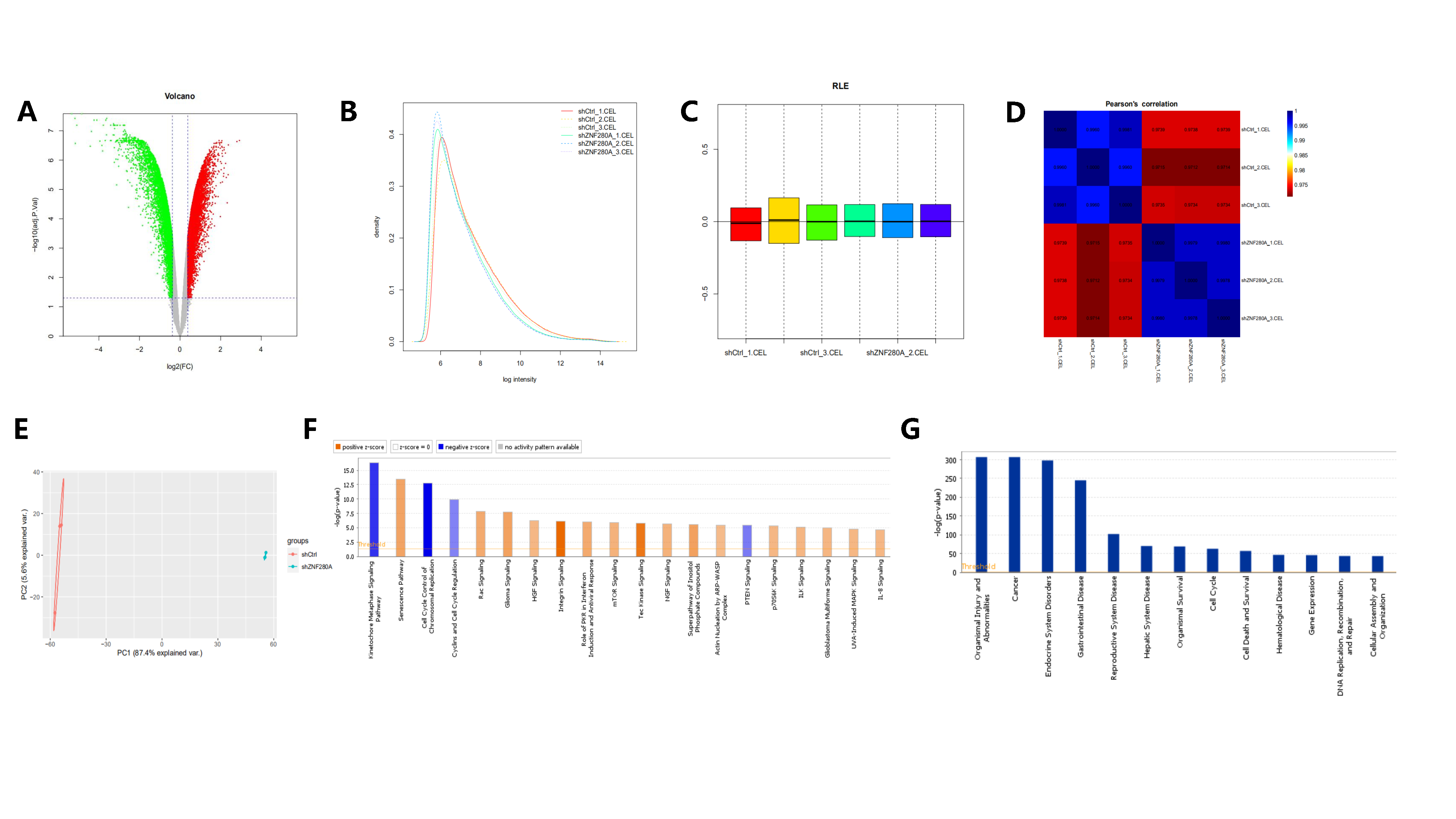

Supplement: Supplementary Figure 2 — (A) The volcano plot of gene expression profiling in RKO cells with or without ZNF280A knockdown. Green dots represented the downregulated DEGs, red dots represented the upregulated DEGs. (B–E) The quality of all the microarray data was assessed in several ways. (F, G) Results showed cell cycle control of chromosomal replication as one of the most enriched pathways and cancer as one of the most enriched diseases. [file Image_2.png]

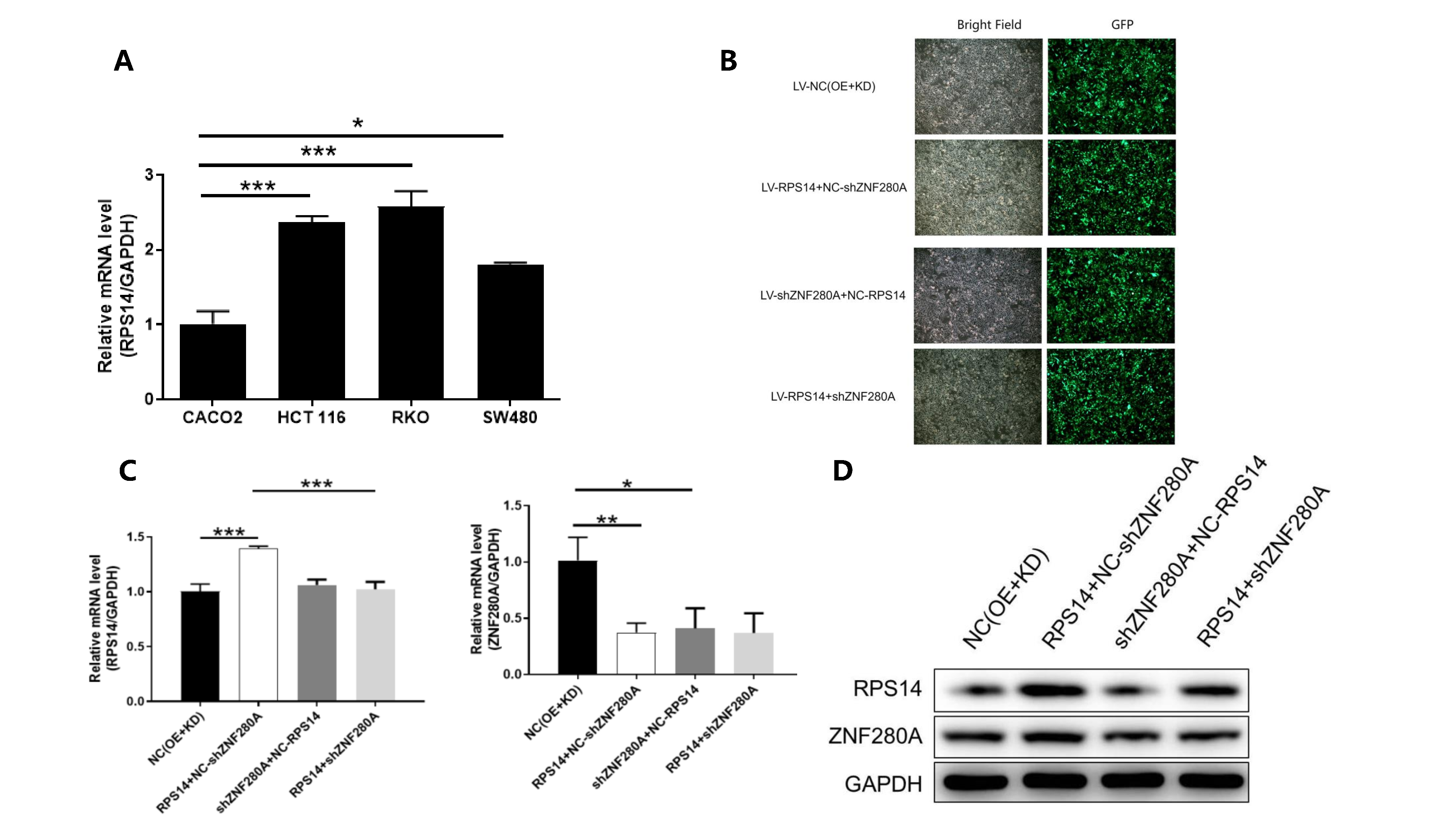

Supplement: Supplementary Figure 3 — (A) The expression of RPS14 in CRC cells was analyzed by qPCR. (B) The transfection efficiencies in RKO cells were evaluated through observing the fluorescence of GFP on lentivirus vector. (C, D) The expression of ZNF280A and RPS14 of the indicated cells were e analyzed by qPCR and WB. *P < 0.05, **P < 0.01, ***P < 0.001. [file Image_3.png]

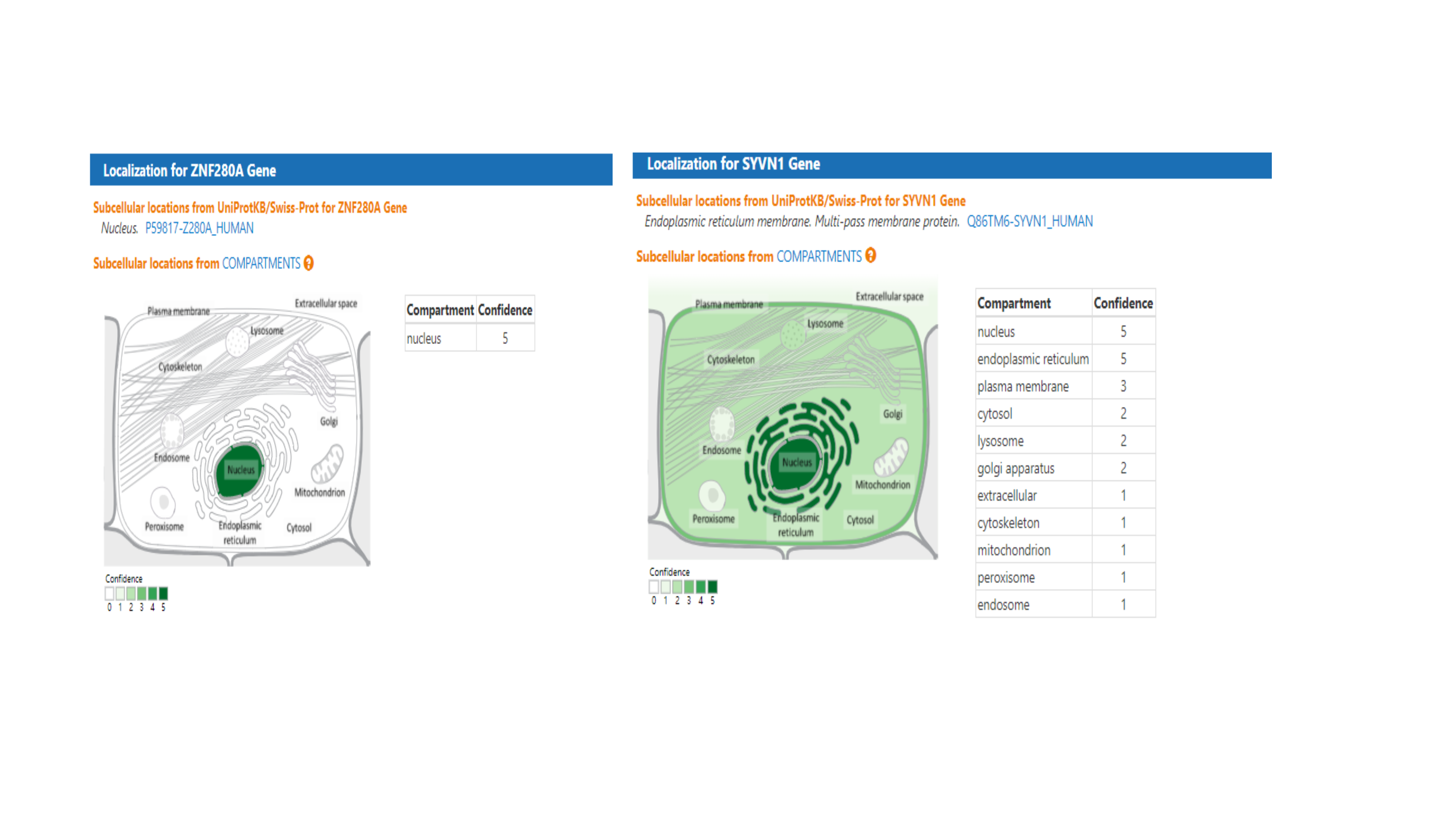

Supplement: Supplementary Figure 4 — Subcellular localization analysis indicated that there was a great possibility of interaction between ZNF280A and SYVN1. [file Image_4.png]
